# Comparative Multi-Epitope-Ligand-Cartography reveals essential immunological alterations in Barrett's metaplasia and esophageal adenocarcinoma

**DOI:** 10.1186/1476-4598-9-177

**Published:** 2010-07-06

**Authors:** Uta Berndt, Lars Philipsen, Sebastian Bartsch, Yuqin Hu, Christoph Röcken, Wiedenmann Bertram, Marcus Hämmerle, Thomas Rösch, Andreas Sturm

**Affiliations:** 1 Department of Medicine, Division of Gastroenterology and Hepatology, Charité-Campus Virchow Clinic, Universitätsmedizin Berlin, Germany; 2 MelTec GmbH & Co. KG, Magdeburg, Germany; 3 Institute of Pathology, Christian-Albrechts-University, Kiel, Germany; 4 Institute for Molecular and Clinical Immunology, Otto-von-Guericke-University Magdeburg, Germany; 5 Department of Interdisciplinary Endoscopy, University Medical Center Hamburg-Eppendorf, Hamburg, Germany

## Abstract

**Background:**

Barrett's esophagus (BE) is caused by gastroesophageal reflux with consecutive mucosal inflammation, predisposing patients to the development of esophageal adenocarcinoma (EAC). We investigated changes in T cell-related mucosal combinatorial molecular protein patterns in both diseases using the novel Multi-Epitope-Ligand-Cartography, a unique robotic whole-cell imaging technology that simultaneously visualizes dozens of proteins in structurally intact tissues and correlates cellular localization of proteins with function.

**Results:**

Biopsies were taken during endoscopy from BE, EAC, and normal control tissue, and proteomic microscopy was performed on 32 different epitopes. When the significance level was set to p < 0.0005 and the search depth to five antibody combinations, controls and BE can be differentiated by 63, controls and EAC by 3222, and BE from EAC by 1521 distinct protein combinations.

For example, the number of activated apoptotic naïve and memory T cells was significantly increased only in BE, whereas the number of activated apoptotic helper and regulatory T cells was significantly elevated in BE and EAC. In contrast, the number of activated apoptotic cytotoxic T cells was significantly elevated only in EAC. Confirming different pathways in BE and EAC, the number of T lymphocytes with p53 expression and downregulation of bcl2 expression (CD3^+^p53^+^Bcl2^-^NfkB^-^) was significantly increased in EAC compared to BE and controls. Interestingly, the number of precursor T cells (CD7^+^) was significantly elevated only in EAC. These cells lack Bax and caspase-8, suggesting impaired apoptosis in the early stages of T cell differentiation.

**Conclusion:**

Proteomic analysis showed for the first time that proteins, which are critically involved in the mucosal immune system of the esophagus, are distinctly expressed in BE and EAC, whereas others are comparably altered in both diseases, suggesting that many pathogenic events might be shared by both diseases. Topological proteomic analysis, therefore, helps us to understand the different pathogenic events in the underlying disease pathways.

## Background

In the recently updated guidelines Barrett's esophagus is defined as endoscopically apparent displacement of the squamocolumnar junction proximal to the gastroesophageal junction with histopathological confirmation of intestinal metaplasia characterized by goblet cells [1,2]. It is widely accepted that metaplasia develops as a consequence of gastro-esophageal reflux disease and may cause esophageal adenocarcinoma via progression of low- and high-grade intraepithelial neoplasia [3-5]. But in contrast to the relatively high incidence of reflux symptoms of 10-20% in the Western population [6], the prevalence of Barrett's metaplasia in patients undergoing an upper endoscopy for gastroesophageal reflux disease is only about 10% [7,8]. Despite the comparatively low annual incidence rate for developing an esophageal adenocarcinoma of approximately 0.5% for patients with BE [9], researchers focus on the mechanisms involved in the metaplasia-dysplasia-carcinoma sequence because of the poor prognosis of this adenocarcinoma. However, despite the fact that inflammation is a critical component of tumor progression [10], current knowledge of the molecular mechanisms of EAC carcinogenesis on a cellular level is largely limited to the role of epithelial cells. Since T cells trigger inflammation, their distribution and function needs to be investigated to provide a better understanding of EAC carcinogenesis. The cellular environment and spatial arrangements of T cells determines their function. Therefore analyzing the phenotype of cellular components in morphologically intact fixed tissue is a promising approach for uncovering distinct protein expression patterns that are potentially critically involved in cancer development.

The novel Multi-Epitope-Ligand Cartography technology has been used by our group and others to perform systematic high-content proteomic analysis of colorectal cancer [11], psoriasis [12], murine hippocampus [13], Crohn's disease, and ulcerative colitis [14]. With the creation of a highly flexible multiplex detection system the use of fluorescent *in situ *protein detection has been improved. This unique robotic whole-cell imaging technology is able to simultaneously visualize dozens of proteins in structurally intact cells or tissue. The highly complex information generated by MELC is processed through advanced data analysis and visualization software to identify protein networks that play a crucial role in biological processes. The advantage of using a multidimensional microscopic robot technology for high-throughput protein recognition allows us to detect the considerable amount of 4.3 × 10^9 ^protein expression arrays and enables the generation of a protein collocation map, which can be summarized as a toponome.

Our study represents the first systematic, *in situ *investigation of T cell-related protein expression patterns and their modification in the tissue of BE and EAC patients. By comparing the protein expression pattern of healthy controls with that of BE and EAC, we ultimately aimed to reveal changes in the expression and distribution of key proteins which in the multistep sequence from normal squamous epithelium via Barrett's metaplasia may finally lead to esophageal adenocarcinoma.

## Results

### Exploring combinatorial molecular phenotypes within the esophageal mucosa in BE, EAC and controls

We previously confirmed the reproducibility and robustness of the MELC technology to adequately investigate protein expression patterns of the intestinal mucosa [14]. Given the advantages of this technology for exploring not only single markers, but also complex protein expression patterns, our first question of general interest focused on the absolute number of significant different combinatorial molecular phenotypes (CMP). When the significance level was set to p < 0.0005 (t-test) and the search depth to mostly five antibody combinations, controls and BE can be differentiated by 63, but controls and EAC by 3222, and BE from EAC by 1521 distinct protein combinations. Further analysis of the margin of the respective differences revealed that, within the control vs. EAC comparison, most CMPs displayed threefold and tenfold differences whereas a comparison of normal vs. BE shows only 10 CMPs that were tenfold increased in Barrett's esophagus (Table 1).

**Table 1:** Numeric distribution of differentially expressed CMP motifs in control, BE and EAC

	3-fold up	10-fold up	3-fold down	10-fold down
Control vs. BE	47	10	0	0

Control vs. EAC	2483	1056	232	99

BE vs. EAC	807	219	201	43

The given values are the total numbers of significantly up- and down-regulated expression of combinatorial molecular phenotype motifs within the two groups (p < 0.0005).

### Mucosal T-cell distribution in the esophagus

**Figure 1:**
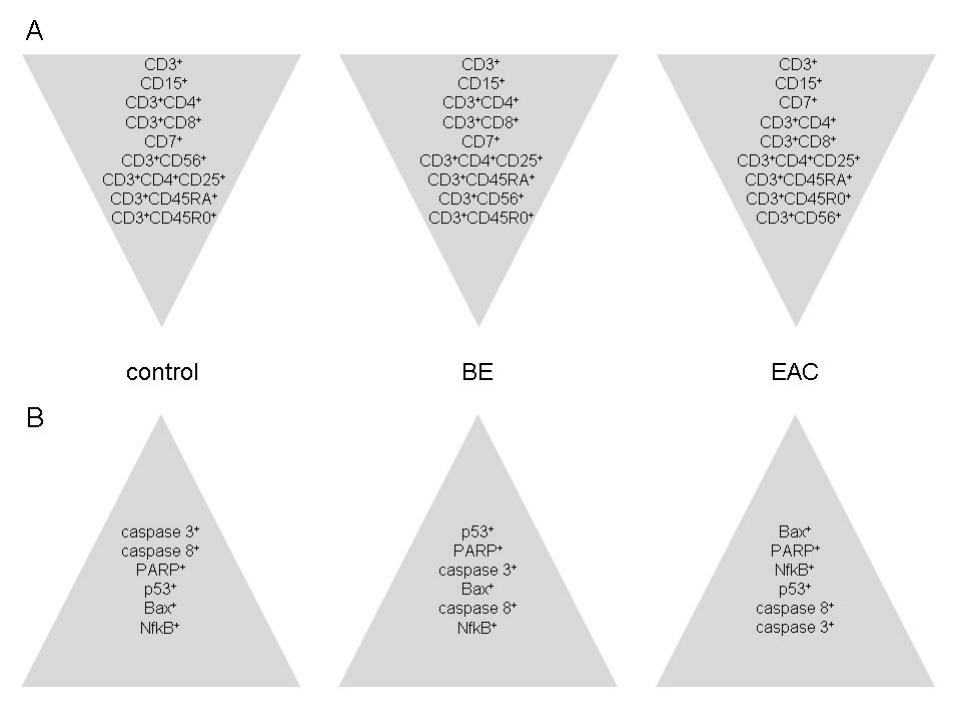
**Schematic demonstration of the T-cell distribution and expression of apoptotic markers including NfkB within the esophageal mucosa in control, BE and EAC**. *A *T-helper cells are the most frequent T cells in control and BE, whereas in EAC T precursor cells are the most frequent T cells. The position of natural killer cells (CD56^+^) decreases from controls via BE to the last position of EAC. *B *The relative frequency of central apoptotic markers as well as p53 and NfkB varies between control, BE and EAC. Biopsies were taken from control, BE, and EAC mucosa and MELC staining cycles, and data processing was performed as described in the Experimental Procedures section.

Aiming to define the T-cell populations within the distal esophagus, we evaluated the relative frequency of T-cell phenotypes in the three entities. Interestingly, T-helper cells (CD4^+^) were the most present T cells in the mucosa of control and BE, whereas precursor T lymphocytes (CD7^+^) are the most frequent T cell population in EAC (Figure 1a). Also, the ranking of natural killer cells (CD56^+^) decreases from 6^th ^position in controls to 8^th ^position in BE to the last position in EAC. It was noted that, next to T cells, neutrophiles (CD15^+^) were comparably expressed in all three entities.

### Apoptotic activity and NfkB expression within the esophageal mucosa

Given the fact that dysregulation of apoptosis and cell activation are central hallmarks of carcinogenesis [15,16], we went on to analyze the relative distribution of apoptotic cell markers as well as NfkB protein expression as a marker of cell activation within the esophageal mucosa of the three groups. As shown in Figure 1b not only NfkB, but also the distribution of centrally important apoptotic markers as well as p53 significantly varied between controls, BE, and EAC.

### 31 most frequent CMPs are distinct in control, BE and EAC

**Figure 2:**
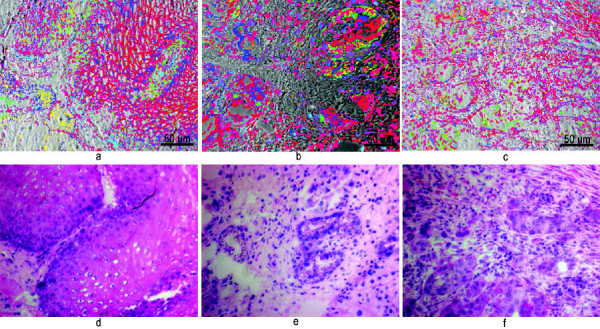
**Visualization of the 31 most frequently CMPs in control (*A*), BE (*B*) and EAC (*C*) in a phase contrast image**. Each of the 31 different colors represents a specific CMP consisting of protein expression signals and lack of protein expression signals. Biopsies were taken from control, BE, and EAC mucosa, and MELC staining cycles and data processing were performed as described in the Experimental Procedures section. For the respective color legend see Additional File 3, Table S2. Figure 2 *D-F *shows the respective H&E staining for each overlay (magnification 200×) Scalebar 50 μm

The results so far uncovered significant differences in protein expression profiles in controls, BE, and EAC and thus, using the MELC technology, we visualized the 31 most frequently expressed CMPs (Figures 2a-c). Obvious differences between the images confirmed the distinct protein expression patterns between the three groups, prompting us to uncover the respective combinatorial molecular phenotypes in detail.

### Distinct T cell CMP expression in control, Barrett and EAC mucosa

The important role of inflammation in the development of cancer is widely accepted [10], and the function of T lymphocytes play a central role within this process. Our analysis revealed that although the relative frequency of the single marker CD3^+ ^is comparable in all groups (data not shown), the co- and anti-localization of further markers uncovered significant differences of T-cell death pathways between controls, BE, and EAC.

**Figure 3:**
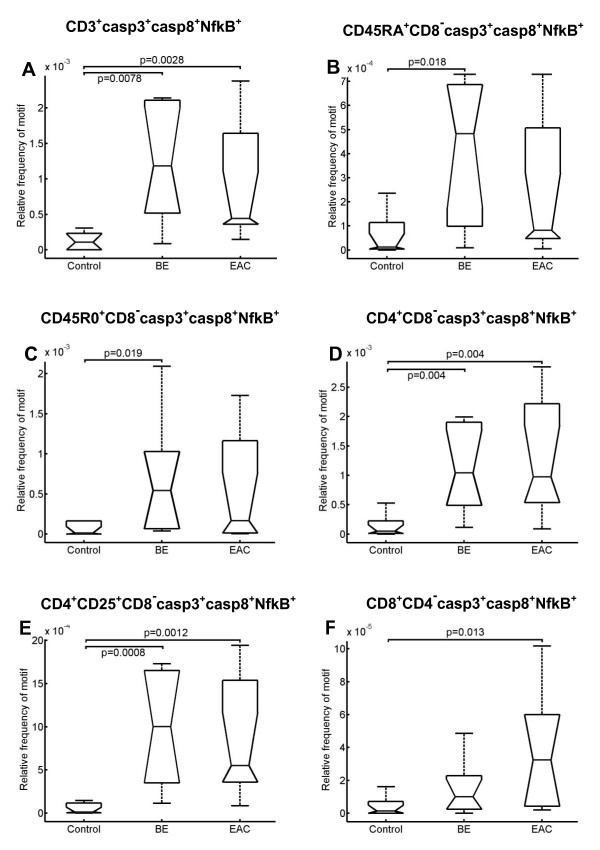
**Boxplot analysis of the apoptotic activity of different T-cell populations**. *A*, The number of apoptotic T cells is significantly increased in BE and EAC compared to the controls. *B*, *C*, The number of apoptotic naïve T cells as well as the number of apoptotic memory T cells is significantly increased in BE compared to the controls. *D*, *E*, The number of apoptotic T-helper cells as well as apoptotic regulatory T cells is significantly increased in BE and EAC compared to the controls. *F*, The number of apoptotic cytotoxic T cells is significantly increased in EAC compared to the controls. Biopsies were taken from control, BE, and EAC mucosa and MELC staining cycles and data processing were performed as described in the Experimental Procedures section. The respective significance levels are depicted within the graphs.

As depicted in Figure 3a, the number of activated (NfkB^+^) T cells (CD3^+^) with expression of caspase 3 and caspase 8 is increased in BE and EAC compared to the controls. Further investigations showed that apoptotic naïve T cells (CD45RA^+^CD8^-^caspase3^+^caspase8^+^NfkB^+^) and apoptotic memory T cells (CD45R0^+^CD8-caspase3^+^caspase8^+^NfkB^+^) are increased in BE compared to the controls and EAC (Figure 3b and 3c). In contrast, apoptotic T-helper cells (CD4^+^CD8-caspase3^+^caspase8^+^NfkB^+^) and regulatory T cells (CD4^+^CD25^+^CD8-caspase3^+^caspase8^+^NfkB^+^) are increased in both BE and EAC (Figure 3d and 3e). However, the number of apoptotic cytotoxic T cells (CD8^+^CD4-caspase3^+^caspase8^+^NfkB^+^) is significantly elevated only in EAC (Figure 3f).

**Figure 4:**
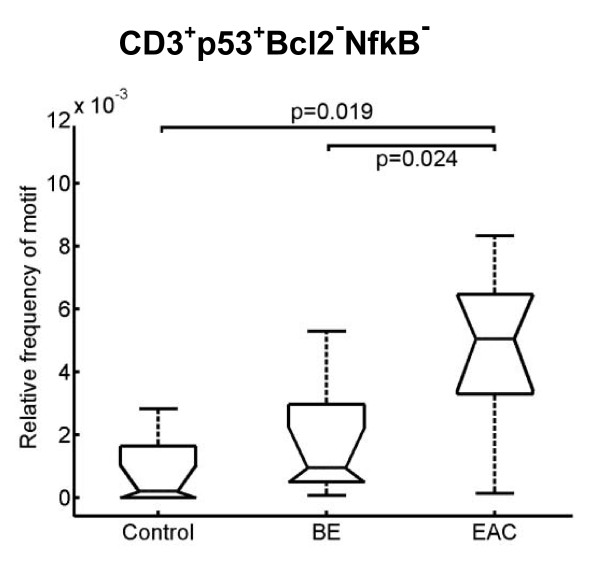
**Boxplot analysis of CMPs containing p53 expression signals in T cells**. The number of T cells with expression of p53 and downregulation of Bcl2 und NfkB is significantly increased in BE and EAC compared with the controls. Biopsies were taken from control, BE, and EAC mucosa and MELC staining cycles and data processing were performed as described in Experimental Procedures. The respective significance level is depicted within the graph.

Although p53 is a key player in carcinogenesis [17], its expression in T-helper cells (CD4^+^), cytotoxic T cells (CD8^+^), naïve T cells (CD45RA^+^) as well as memory T cells (CD45R0^+^) was comparable in all groups (data not shown). However, the number of CD3^+^p53^+^Bcl2-NfkB^- ^cells was increased in EAC compared to BE and the controls (Figure 4).

**Figure 5:**
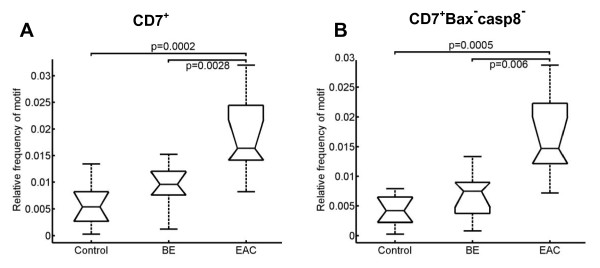
**Boxplot analysis of precursor T cells**. *A*, The number of precursor T cells in BE and EAC is significantly increased in BE and EAC compared to the controls. *B*, The number of precursor T cells without apoptotic activity is increased in BE and EAC compared to the controls. Biopsies were taken from control, BE, and EAC mucosa and MELC staining cycles and data processing were performed as described in the Experimental Procedures section. The respective significance levels are depicted within the graphs.

**Figure 6:**
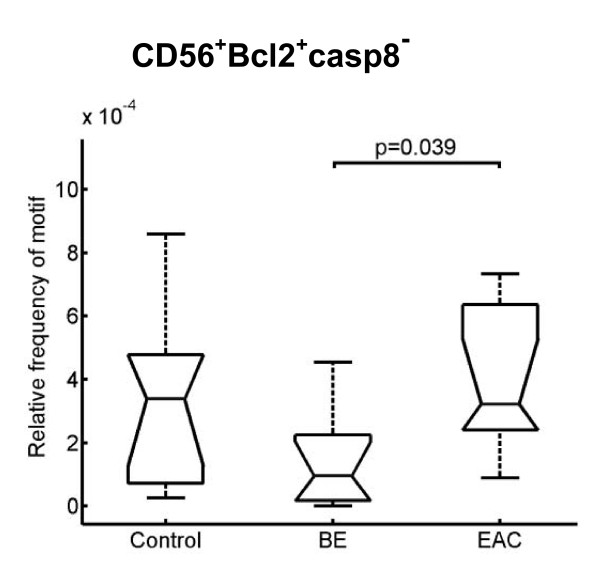
**Boxplot analysis of natural killer cells**. The number of antiapoptotic natural killer cells is significantly decreased in BE compared to EAC. Biopsies were taken from control, BE, and EAC mucosa and MELC staining cycles and data processing were performed as described in the Experimental Procedures section. The respective significance level is depicted within the graph.

As expected, the highest number of CMP motifs out of the 4.3 × 10^9 ^possible CMP motifs exists between the control group and EAC. Exploring which proteins are distinctly regulated in the progression from BE to EAC, we examined the motifs containing precursor T lymphocytes (CD7^+^) and natural killer cells (CD56^+^) based on the change in their relative frequency within the three entities as stated above. The number of CD7^+ ^cells was significantly increased in EAC compared to BE and the controls (Figure 5a). Colocalization with apoptotic markers showed a significant difference for CD7^+ ^cells lacking Bax and caspase 8 expression (Figure 5b). This failure of apoptotic activity was also observed in natural killer cells (CD56^+^), where the number of CD56^+ ^cells expressing Bcl2 and lack of caspase 8 expression was significantly decreased in BE compared to EAC (Figure 6).

## Discussion

Induction of Barrett's metaplasia and its malignant transformation resulting in esophageal adenocarcinoma has been a field of great interest since the incidence of these two disease entities has increased over the last 30 years [18-20]. The majority of research topics embracing the molecular biology of BE and EAC focuses on genetic alterations, which are possibly caused by various genetic, epigenetic and microenvironmental factors [4,21]. Furthermore, the existing histopathological data on esophageal inflammatory reactions were described in peptic [22] or eosinophilic esophagitis [23] or analyzed proteins, e.g cytokines, from the complete mucosa, [24-26]. Despite the strong functional association between chronic inflammation and carcinogenesis [10,27] a broad systematic analysis of the cell populations causing inflammatory reaction in the terms of BE and EAC compared to the healthy esophagus was still missing.

In this study we applied the MELC technology, a unique and novel technology for high-throughput protein recognition, which enables the investigation of complex protein expression patterns. The main advantages of the MELC technology over conventional immunohistology are its capacity to analyze co-localisation of proteins and missing protein expression, the so-called anti-localization code. This unique technology allows the creation of a complete proteome of the tissue. Furthermore, the fact that a whole tissue section can be analyzed, rather than just the partial reproduction of cell activity interactions allowed by conventional methods, enables greater objectivity.

Based on the antibody library and the search depth of five antibodies we identified 4806 different combinatorial molecular patterns within normal controls, BE and EAC out of 4.3 × 10^9 ^possible patterns. We found the lowest number of distinct CMP motifs in the group controls vs. BE. Analyzing these findings in more detail by using nine different single cell markers for T cells including neutrophiles we revealed that healthy controls and BE shared T-helper cells as the most common cell population, explaining the formerly described equal cytokine profiles of non-inflamed squamous esophagus and BE by Fitzgerald et al. [25].

In contrast, the highest number of significant different CMP motifs was found in the group controls vs. EAC. Indeed, exploring the T-cell distribution in EAC compared to the controls and BE, the position of the T-cell subpopulations was different.

T cells have the ability to recognize oncogenic precursor signals and thus prevent malignant tissue transformation [28]. In normal, resting T cells NfkB is rarely constitutively expressed. It is upregulated upon activation [29]. T cells need to be activated and first enter the cell cycle before undergoing apoptosis [30,31]. Thus, with the aim of investigating the role of T cells in the transformation from normal to malignant tissue in the esophagus, we determined the expression of pro-apoptotic signals together with NfkB^+^. In BE and EAC, activated T cells have a significantly higher caspase expression and, in turn, a higher apoptosis rate than the controls, suggesting that in the malignant transformation, immune control mechanisms are compromised. This finding is further supported by the increased inability of the immune system to control local immune homeostasis, as in EAC regulatory (CD4^+^CD25^+^), and effector (CD8^+^) T cells appear to have a higher death rate.

The large amount of CD7 positive cells - which are ranked before T-helper cells as one of the most important cell populations of the adaptive immune system - in EAC was even elevated to a significant level compared to BE. CD7 is considered to be one of the earliest surface markers in T-cell ontogeny [32] and can function as a co-stimulatory molecule after T cell activation [33]. In the context of malignant transformation many lymphomas with a lack of CD7 expression have been described to date [34,35]. Thus, the first description of an increased number of progenitor T lymphocytes in EAC should warrant further investigations. In contrast to CD7, the role of natural killer cells (CD56^+^) as primary responders with an ability to recognize and lyse tumor cells without previous sensitization is well known [36]. In our study, despite the increased number of natural killer cells with expression of antiapoptotic Bcl2 and down-regulation of caspase 8, natural killer cells ranked in the last position in EAC. Interpretation of our findings on CD7 and CD56 implies that the low number of CD56 cells can cause up-regulation of progenitor T lymphocytes to enforce an immunological response against the tumor cells.

Given the importance of apoptosis for the homeostatic control of lymphocyte populations [37,38] as well as the knowledge that cancer development is linked with an acquired ability to resist apoptosis [15], we further focused on the apoptotic activity of the T lymphocytes within the esophageal mucosa. Apoptotic activity in the entire T lymphocyte population in BE as well as in EAC was up-regulated. This result confirms our previously published findings of increased apoptotic activity of T cells in colorectal cancer [11]. However, a distinct regulation of apoptotic activity in the various T-cell subpopulations was revealed since the number of apoptotic naïve T cells and apoptotic memory T cells was increased only in BE, whereas the number of apoptotic T-helper cells and apoptotic regulatory T cells was increased in BE and EAC. Due to the differentiation of naïve T cells into effector und memory T cells [39] an increased number of apoptotic naïve T cells may result in a lower number of effector T cells and, in consequence, in a reduced capacity to act against the development of metaplasia.

## Conclusion

In summary, enabled with a novel and unique, multidimensional, fluorescence-based technology, we carried out the first proteomic analysis of the esophageal mucosa in the metaplasia-cancer sequence. By investigating key immune function-related proteins and by *in situ *detection of their modification our study demonstrated that both BE and EAC induce a tremendous modification of protein expression profiles within the esophageal mucosa. We showed that, as a consequence, not only the protein expression at the single cell level is distinct between controls, BE and EAC, but also the quantity of the specific immune-competent cell population within the mucosa changes. The uncovering of distinct and shared pathways by BE and EAC might thereby help us to understand pathogenic events that finally result in EAC.

## Methods

### Patients and sample preparation

A total of 9 biopsies per group (healthy controls, Barrett's mucosa without dysplasia, and esophageal adenocarcinoma) were analyzed. All biopsies derived from the esophageal mucosa and were obtained endoscopically. In cases of Barrett's mucosa, a pinch biopsy was taken from the middle to the proximal part of the Barrett's epithelium, and was cut into two segments, one for investigation by MELC and one for dysplasia grading confirmation. The biopsies were taken from the middle to the proximal part of the Barrett's epithelium. The median age of the patients with BE without dysplasia was 64 years (range 48-89 years), 8 of them were male and 1 was female. 6 patients were taking proton pump inhibitors at the time of tissue sampling. The median age of the patients suffering from EAC was 62 years (range 51-68 years), 8 of them were male and 1 was female. The biopsy was taken at the time of first diagnosis, and thus, no patient had received antitumor therapy. Control patients underwent upper endoscopy for gastric discomfort, but all of them had a macroscopically normal appearance. The median age of the healthy control patients was 59 years (range 36-72 years), 8 of them were male and 1 was female. All histopathological diagnoses were assessed from at least two various, board certified pathologists and at last confirmed by an experienced attending pathologist (C.R.).

Signed informed consent was obtained from each subject. Both, the protocol and the consent form were reviewed and approved by the local ethics committee of the Charité, Berlin, Germany.

Biopsies were snap frozen in liquid nitrogen, stored at -80°C, and prepared for MELC analysis as recently described [14]. Briefly, after embedding the biopsy in Tissue-Tek (Sakura Finetek, Torrance, California, USA) cryosections of 5 μm thickness were sliced and applied on silane coated coverslips. After fixing the tissue with acetone the coverslips were stored at -20°C. In preparation for the MELC procedure the tissue was fixed once again with acetone, rehydrated with Dulbecco's PBS, and non-specific signals were blocked with normal goat serum. After rinsing the sample five times with PBS analysis of the tissue with the MELC technique was started.

### MELC Technology

#### MELC Library

The MELC library consists of 31 fluorescence tags including antibodies, lectins and propidium iodide (PI) as a nucleic acid dye Additional File 1, Table S1), as recently published [14]. The antibodies can be divided into the following main groups: phenotype markers (CD3, CD4, CD8, CD45RA, CD45RO, cytoceratin, collagen IV), markers for inhibition (Bcl2) and promotion (caspase-3, caspase-8, PARP-2, p53, Bax) of apoptosis, integrin expression markers such as CD11a (LFA-1), CD29 (integrin-β1), CD18 (integrin-β2), markers for antigen presentation (CD1a, HLA-DR, HLA-DQ), adhesion molecule markers (CD2 [LFA-2], CD54 [ICAM-1], CD56 [NKH-1], CD58 [LFA-3], CD44), markers which suggest an activated cell status, like CD25 and NfkBp65, and CD57, CD7, CD71, CD15, WGA for other specific epitopes. Systematical MELC calibration runs as well as conventional immunohistochemistry experiments was performed in order to establish the appropriate working dilutions, fluorophore labels, incubation time (15 min) and positions within the MELC run [40,41]. As a negative control IgG1 antibody was used at the beginning of each MELC experiment.

### Automated microscopic image acquisition by Toponome Imaging Cycler^® ^Multi-epitope readout

We used a Toponome Imaging Cycler^® ^MM3 (TIC) produced by MelTec GmbH & Co. KG, Magdeburg, Germany. This technology platform has been described previously [40-42]. In brief: The fully automated microscopic robot consists of three parts: (i) an inverted wide-field fluorescence microscope (Leica DM IRE2; 20× air lens NA 0.7, filter setups: FITC channel Omega Optical XF116-2; PE channel - Omega Optical XF111-2) with a cooled charge-coupled device camera (Apogee KX4, Apogee Instruments Inc., we use 2 times binning) and motor-controlled XY-stage, (ii) a pipetting unit, and (iii) a computer that controls all hardware components of the robot using MelTecs TIC-Control Software.

After loading the TIC with the specimen, the robot performs the multi-epitope readout as a repetitive cyclic process consisting of 4 steps: (1) the incubation of a fluorochrome-labelled antibody and washing the specimen, (2) acquisition of the fluorescence signal and phase contrast image after correction of any displacement, (3) soft bleaching of the fluorescent dye until no fluorescence signal is detectable, and (4) second acquisition of the fluorescence signal (postbleaching image) and phase contrast image for correction purpose. After completion of these four steps the next cycle starts with the next antibody until the whole experimental design is completed.

#### Image processing and data mining

Fluorescence images produced by each tag as well as the postbleaching images were aligned pixel-wise using the corresponding phase contrast images (accuracy +/- 1 pixel) and were corrected for illumination faults using flat-field correction. We correct the background by a subtraction of the postbleaching image from the following fluorescence image. A mask-setting process excludes invalid pixels not belonging to the biological specimen's information. An experienced histologist together with a scientist (Ph.D.) experienced in MELC technology defined the graylevel threshold on 6 selected MELC runs (two per group) in parallel for each marker separately. These predefined thresholds were automatically applied to all corrected fluorescence images of all MELC runs, reducing the data to binary images comprising the on/off information for the corresponding epitopes. An example of the image processing steps is given in Additional File 2, Figure S1. These images were superimposed to construct a matrix of combinatorial molecular phenotypes, representing a binary code of the 32 epitopes' expression in relation to each pixel (900 × 900 nm^2 ^area) of 1024 × 1024 pixels. The further analysis dealt with CMP motifs characterizing corresponding pixels. These CMP motifs are defined as a pixel-related code of 1/0/* ciphering. The 'TopoMiner'/'MotifFinder' software package (MelTec GmbH & Co. KG, Magdeburg, Germany) was used for data-mining, as described by Schubert et al. and Berndt et al. [14,41]. Three pair-wise screens (Wilcoxon rank-sum test p < 0.0005; search depth n = 5) were performed to detect differences on the level of pixel frequencies of the CMP motifs between the three groups (healthy control, BE, EAC).

### Visualization of data of interest

To visualize CMP motifs of interest the 'Topolyzer' (MelTec GmbH & Co. KG, Magdeburg, Germany) was used. This software package allows the visualization of CMP motifs of interest as tables, boxplots [43-45], and toponome maps on the level of fluorescence and binarized images [46]. Colocalization maps were constructed by superimposing a set of illumination-adjusted colorized fluorescence images, each with a distinct color and transparency.

## Abbreviations

BE: Barrett's esophagus; EAC: esophageal adenocarcinoma; CD: cluster of differentiation; MELC: Multi-Epitope-Ligand-Cartography; Bax: Bcl-2 antagonist X; PARP: poly(ADP-ribose)-polymerase; PI: propidium iodide; CMP: combinatorial molecular phenotypes; Foxp3: forkhead box protein P3; WGA: wheat germ agglutinin.

## Competing interests

Lars Philipsen, Sebastian Bartsch and Marcus Hämmerle are employed at MelTec GmbH & Co. KG.

## Authors' contributions

UB and AS designed the experiment, analyzed the data and wrote the manuscript; UB supervised the experiment; LP, SB and MH took a part in the development of MELC and analyzed the data; YH prepared the samples and collected the clinical data; CR did the conventional HE-staining and diagnosed the biopsies, BW reviewed the manuscript, TR collected the samples and designed the manuscript, AS supervised the design of the study and finalized the manuscript. All authors read and approved the final manuscript.

## Supplementary Material

Additional file 1 **Table S1**. Antibody library. Click here for file

Additional file 2 **Figure S1**. Example of the image processing step based on the run, which is demonstrated in figure 2C and figure 2F. Columns A, B, and C show the original (A), corrected (B), and binarized (C) fluorescence image of the respective marker in the same run. Click here for file

Additional file 3 **Table S2**. Color legend figures 2A-C. Click here for file
