# Supplementary material for: Comparative Multi-Epitope-Ligand-Cartography reveals essential immunological alterations in Barrett's metaplasia and esophageal adenocarcinoma
Source: Mol Cancer. 2010 Jul 6;9:177. doi: 10.1186/1476-4598-9-177 (PMC2909181; doi:10.1186/1476-4598-9-177)
Supplement: Additional file 1 — Table S1. Antibody library. [file 1476-4598-9-177-S1.DOC]

| Ab | ISO Type | Clone | Distributor | Concentration | Exposure time [ms] | Filter setup |
| --- | --- | --- | --- | --- | --- | --- |
| Mouse IgG1 | IgG1 | 679.1Mc7 | Beckman Coulter | 1:10 | 5000 | FITC/PE |
| Caspase 3 active | IgG | C92–605 | BD Biosciences | 1:10 | 5000 | FITC |
| PARP-2 | IgG | Polyclonal | Dianova | 1:90 | 5000 | FITC |
| p53 | IgG2b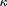 | 80 | BD Biosciences | 1:45 | 5000 | FITC |
| Bax | Polyclonal | Polyclonal | Eurogentec | 1:78 | 5000 | FITC |
| Bcl-2 | Polyclonal | Polyclonal | Eurogentec | 1:88 | 5000 | FITC |
| Caspase 8 | Polyclonal | Polyclonal | Eurogentec | 1:81 | 5000 | FITC |
| NF-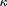B p65 | Unknown | Polyclonal | Biomol | 1:200 | 5000 | FITC |
| CD11a | IgG1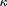 | MHM24 | DakoCytomation | 1:25 | 5000 | FITC |
| CD1a | IgG2a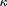 | NA1/34 | DakoCytomation | 1:100 | 5000 | FITC |
| CD2 | IgG2a | 39C1.5 | Beckman Coulter | 1:10 | 5000 | FITC |
| CD25 | IgG2a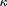 | 7G7B6 | Axxora | 1:100 | 5000 | FITC |
| CD4 | IgG1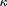 | Coulter clone T4 | Beckman Coulter | 1:10 | 5000 | FITC |
| CD8 | IgG1 | B9.11 | Beckman Coulter | 1:10 | 5000 | FITC |
| HLA-DR | IgG1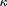 | Immu-357 | Beckman Coulter | 1:50 | 5000 | FITC |
| HLA-DQ | IgG2b | HK19 | Sigma-Aldrich | 1:10 | 5000 | FITC |
| CD45RA | IgG1 | ALB11 | Beckman Coulter | 1:10 | 5000 | FITC |
| CD57 | IgM | NC1 | Beckman Coulter | 1:10 | 5000 | FITC |
| CD54 | IgG1 | 84H10 | Beckman Coulter | 1:10 | 5000 | FITC |
| CD56 | IgG2b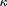 | NCAM16.2 | BD Biosciences | 1:10 | 5000 | FITC |
| CD7 | IgG2a | 8H8.1 | Beckman Coulter | 1:10 | 5000 | FITC |
| CD58 | IgG2a | AICD58 | Beckman Coulter | 1:10 | 5000 | FITC |
| CD71 | IgG1 | YDJ.1.2.2. | Beckman Coulter | 1:50 | 5000 | FITC |
| CD29 | IgG1 | 4B7R | Biozol | 1:10 | 5000 | FITC |
| CD44 | IgG1 | J-173 | Beckman Coulter | 1:200 | 5000 | FITC |
| CD18 | IgG2b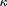 | YFC118.3 | Biozol | 1:10 | 5000 | FITC |
| CD45RO | IgG2a | UCHL1 | Biozol | 1:50 | 5000 | FITC |
| CD3 | IgG1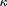 | UCTH1 | BD Biosciences | 1:50 | 5000 | FITC |
| Cytoceratin | IgG1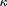 | MNF116 | DakoCytomation | 1:10 | 5000 | FITC |
| Collagen type IV | IgG | Unknown | Hölzel | 1:100 | 500 | FITC |
| CD15 | IgM | AHN1.1 | Axxora | 1:40 | 5000 | PE |
| Propidium iodide |  |  |  | 1:100 | 250 | PE |
| WGA |  |  |  |  | 500 | FITC |
