# Supplementary figures and images for: Comparative Multi-Epitope-Ligand-Cartography reveals essential immunological alterations in Barrett's metaplasia and esophageal adenocarcinoma
Source: Mol Cancer. 2010 Jul 6;9:177. doi: 10.1186/1476-4598-9-177 (PMC2909181; doi:10.1186/1476-4598-9-177)

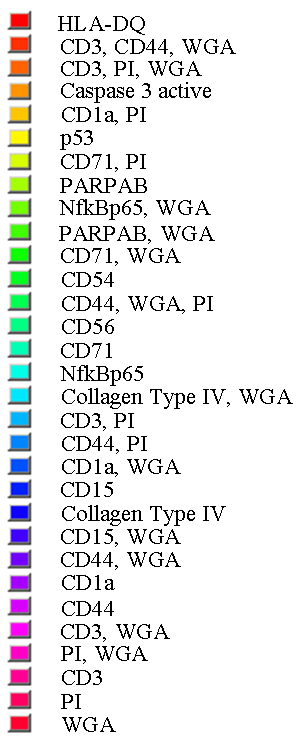

Supplement: Additional file 3 — Table S2. Color legend figures 2A-C. [file 1476-4598-9-177-S3.DOC]
